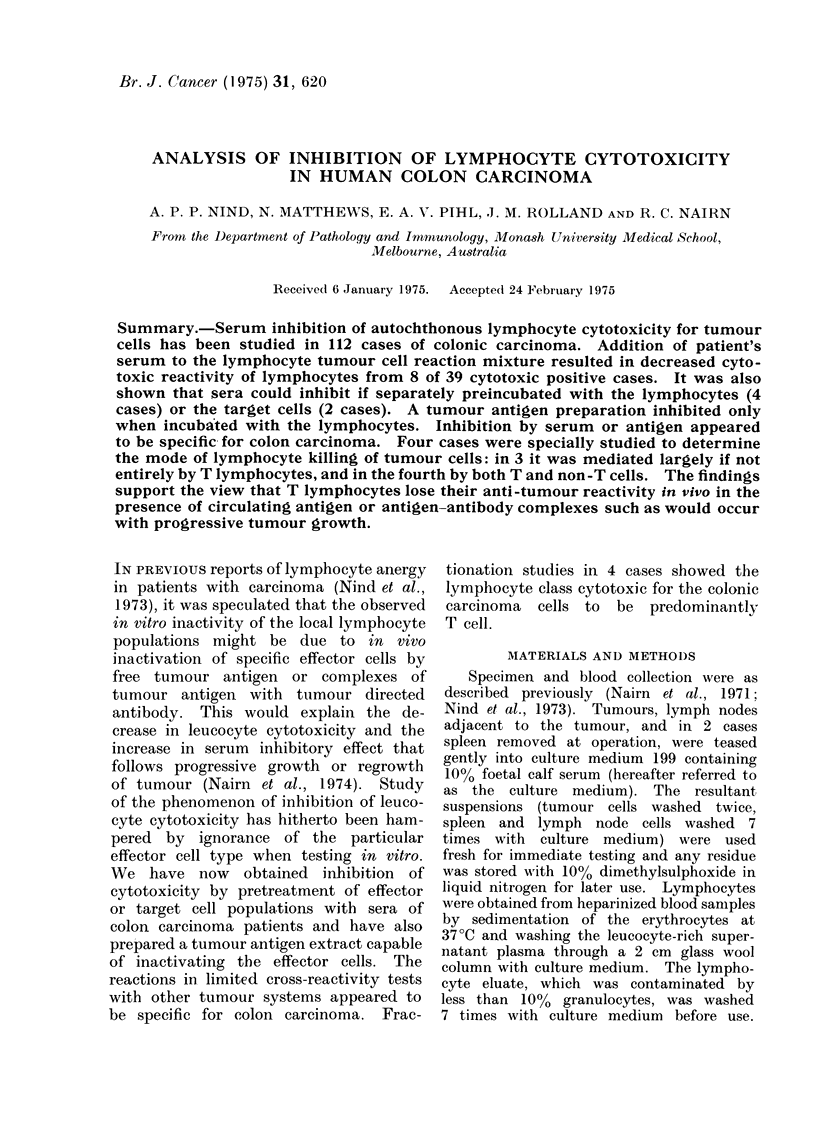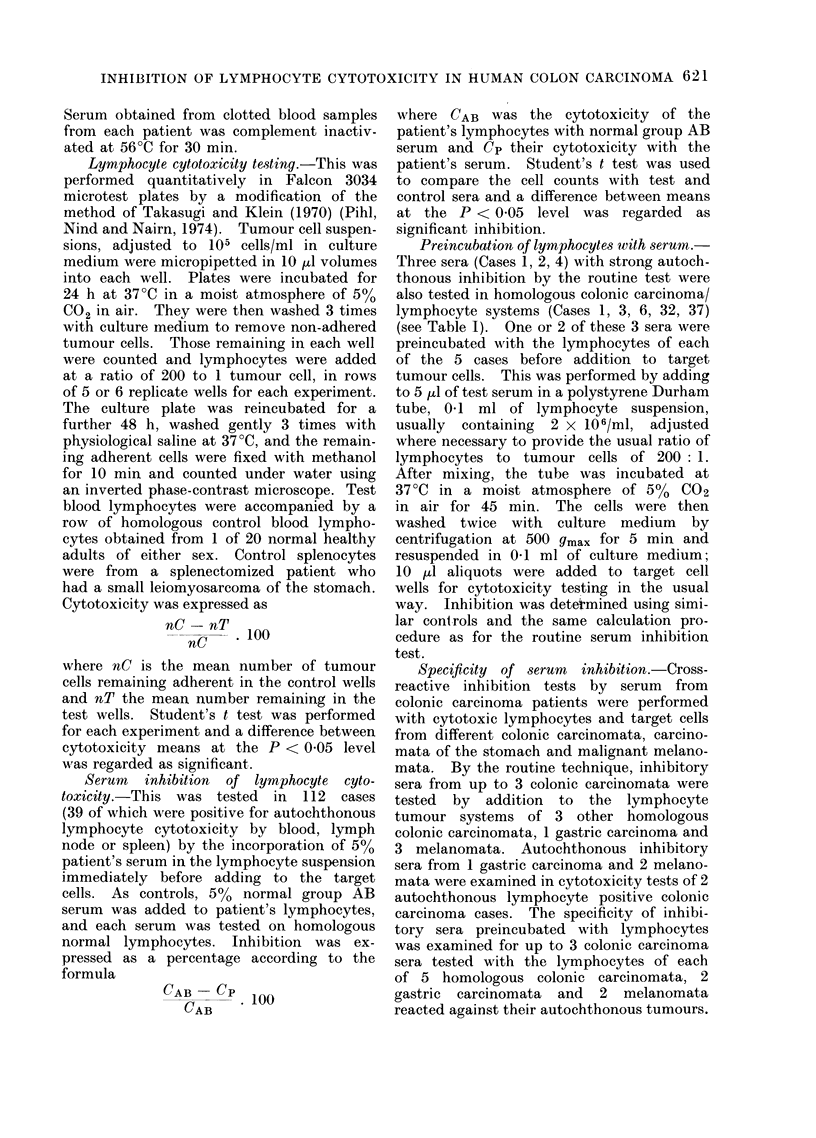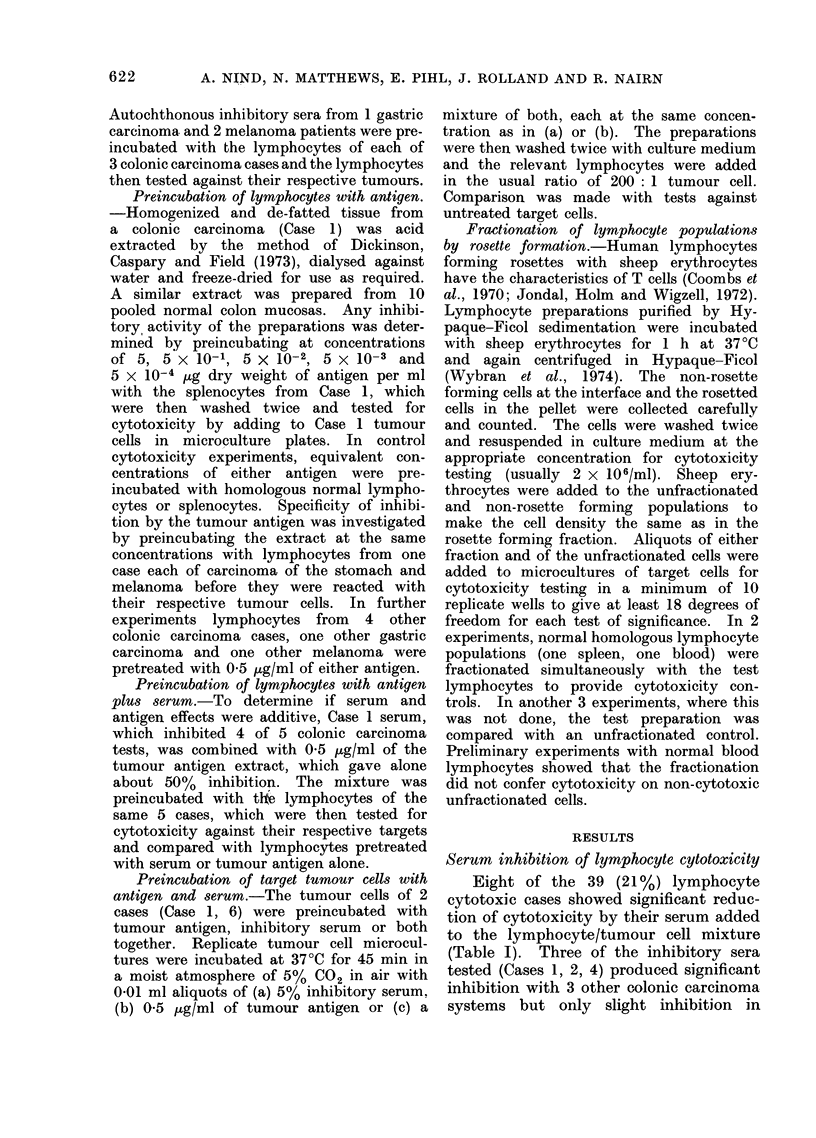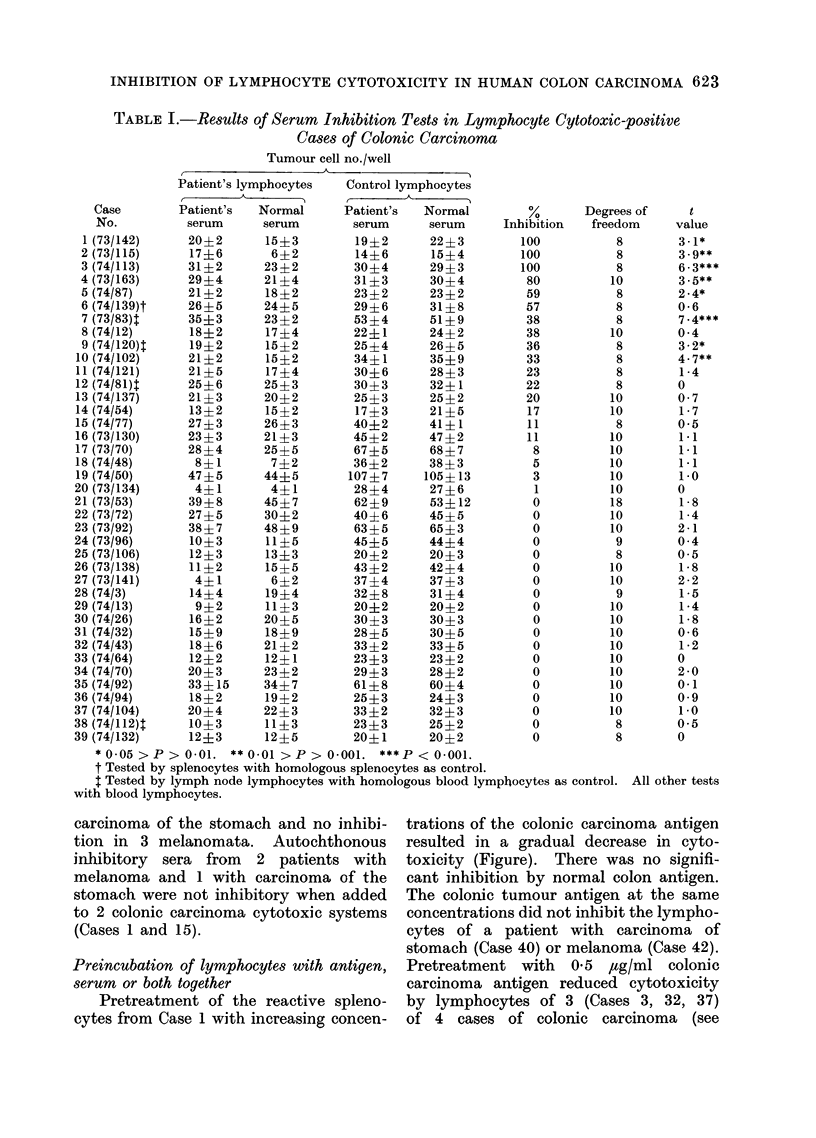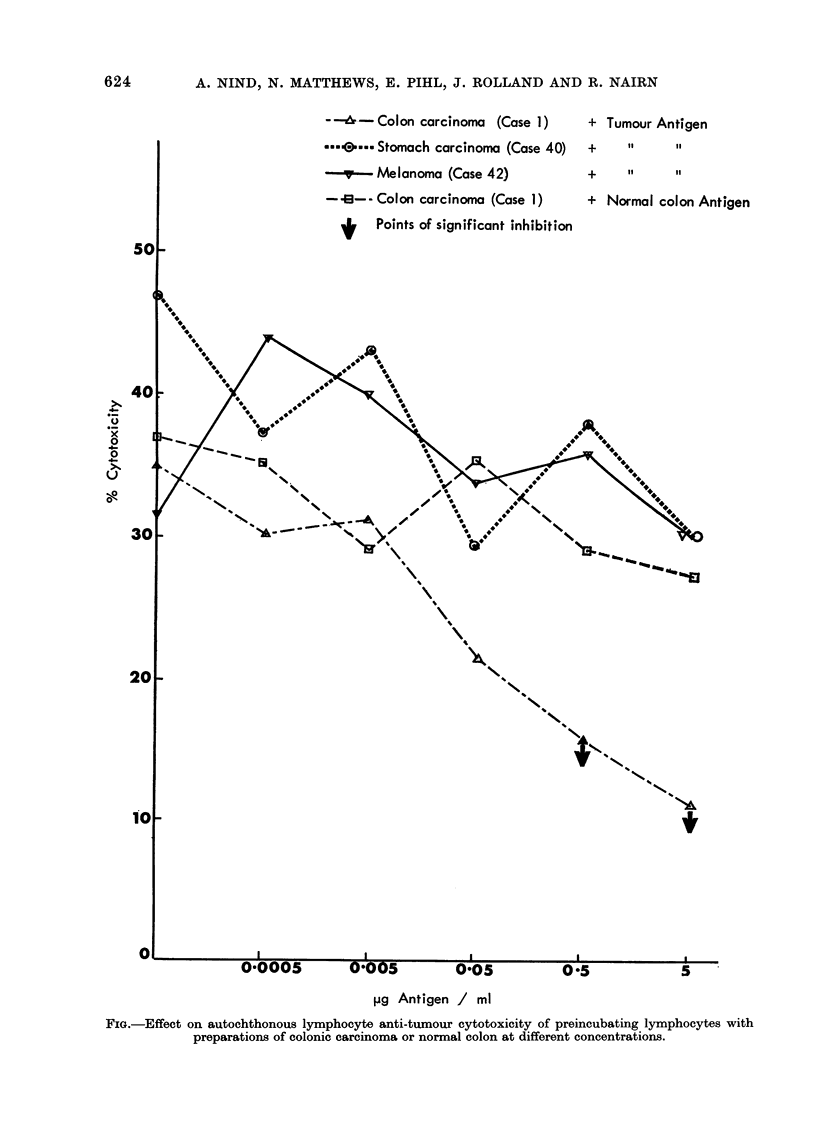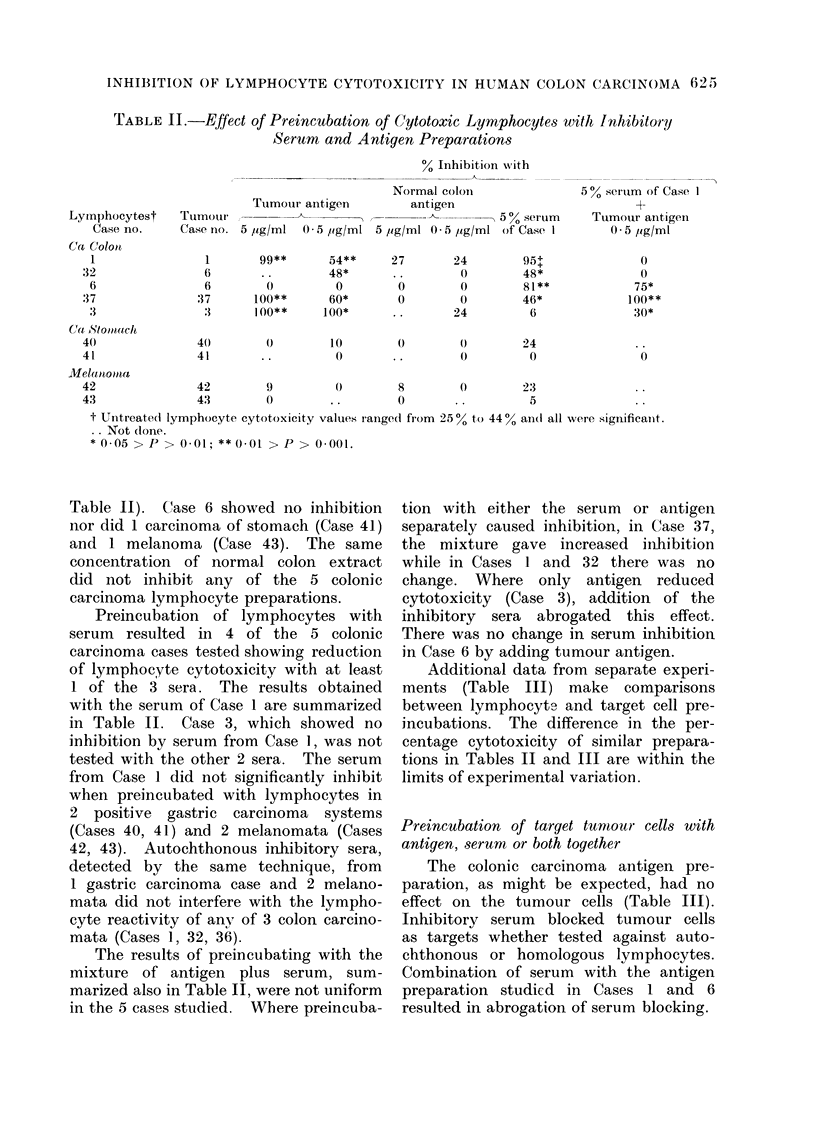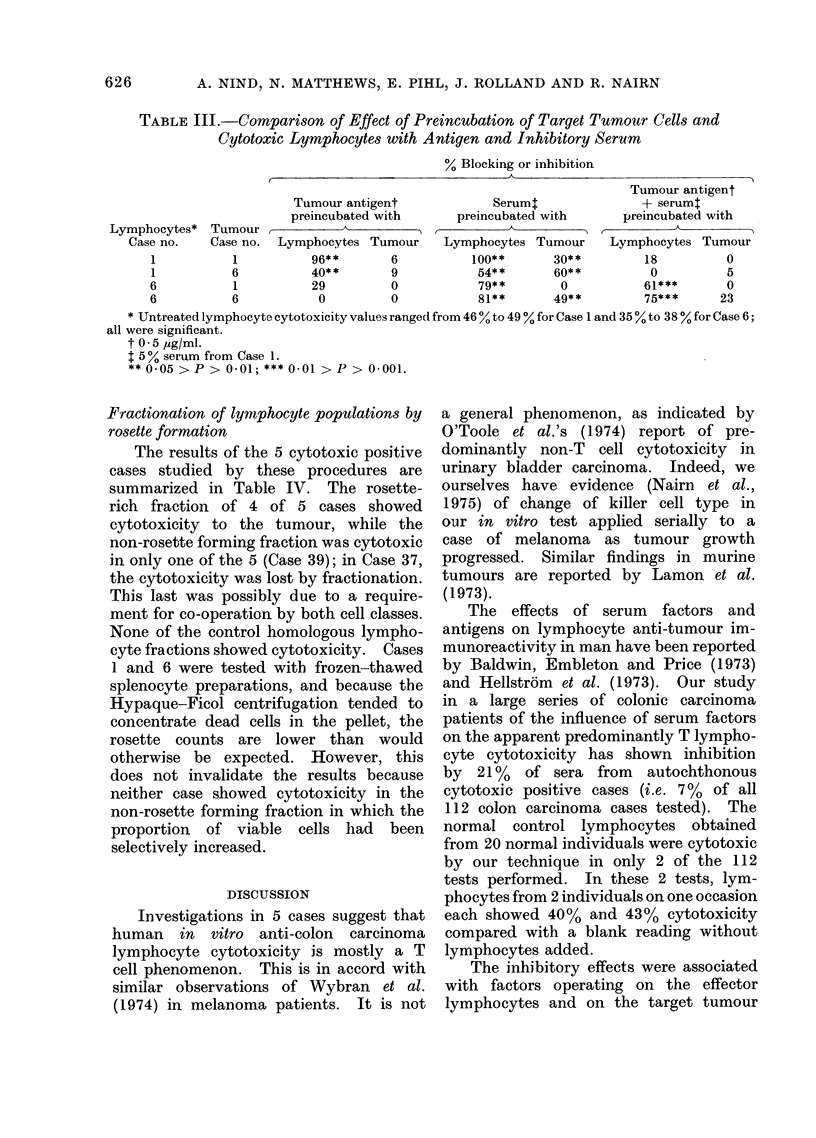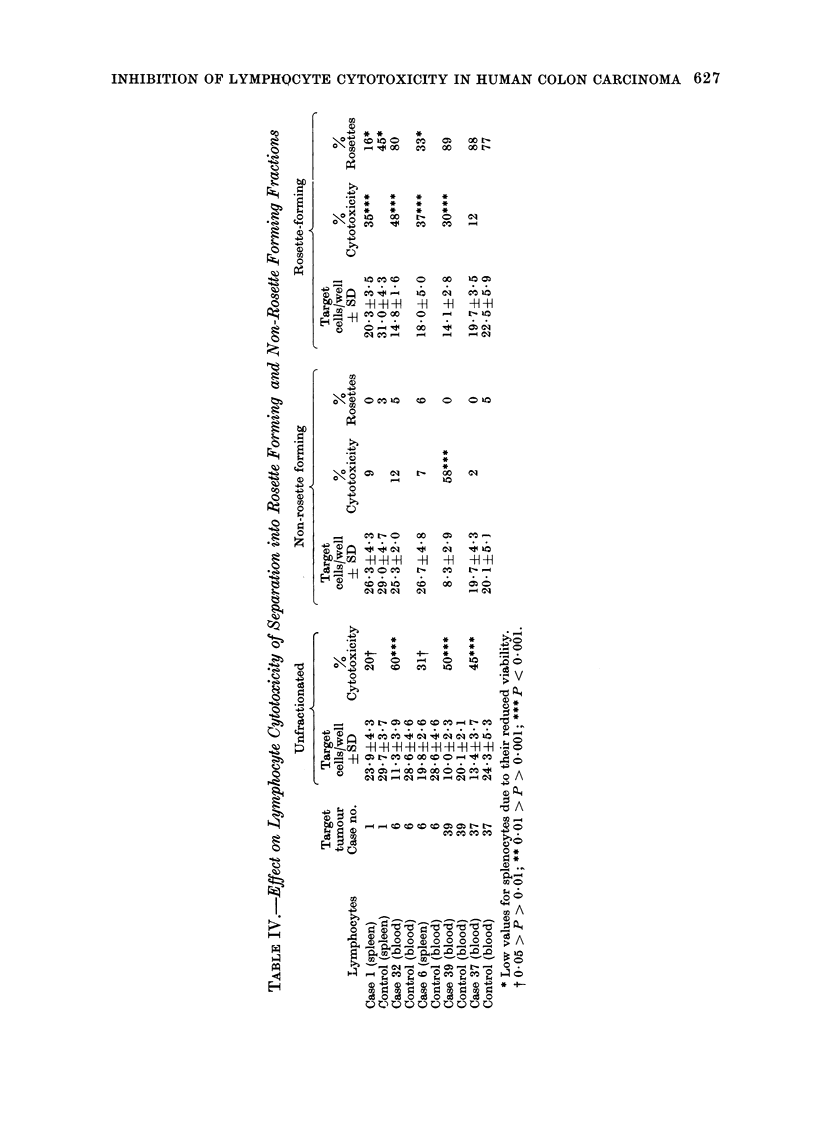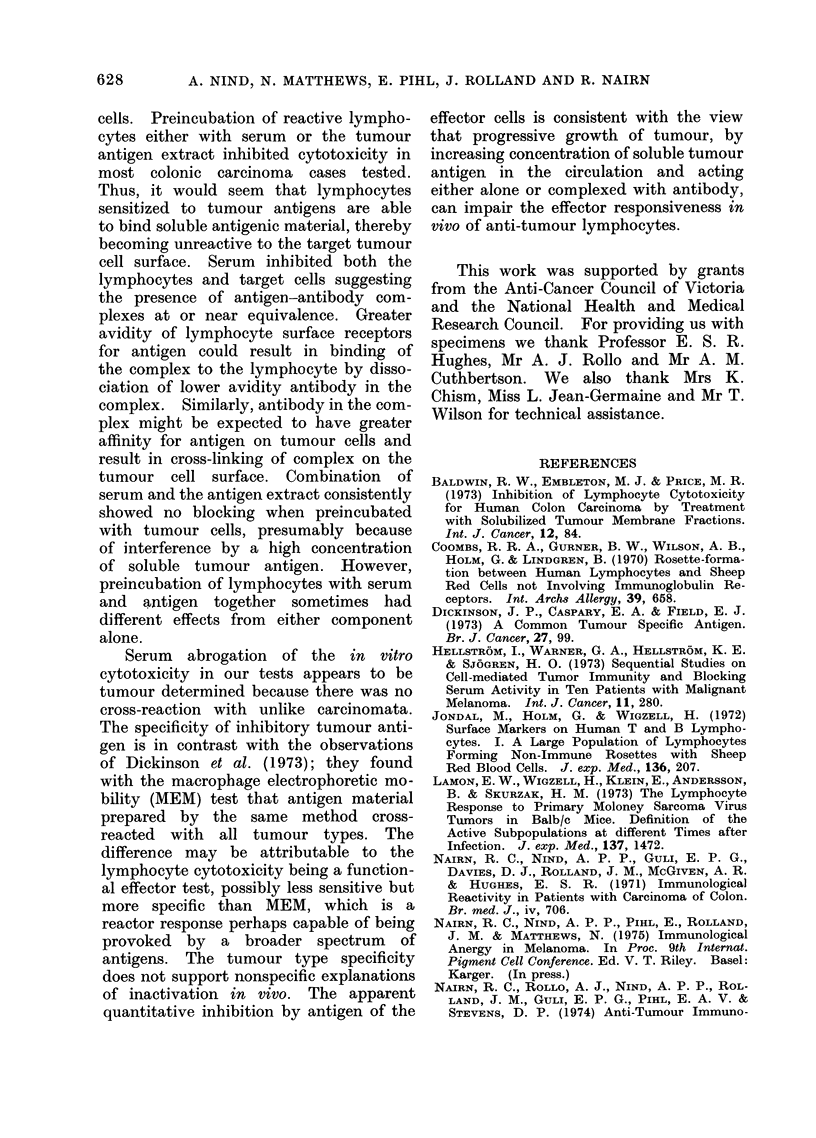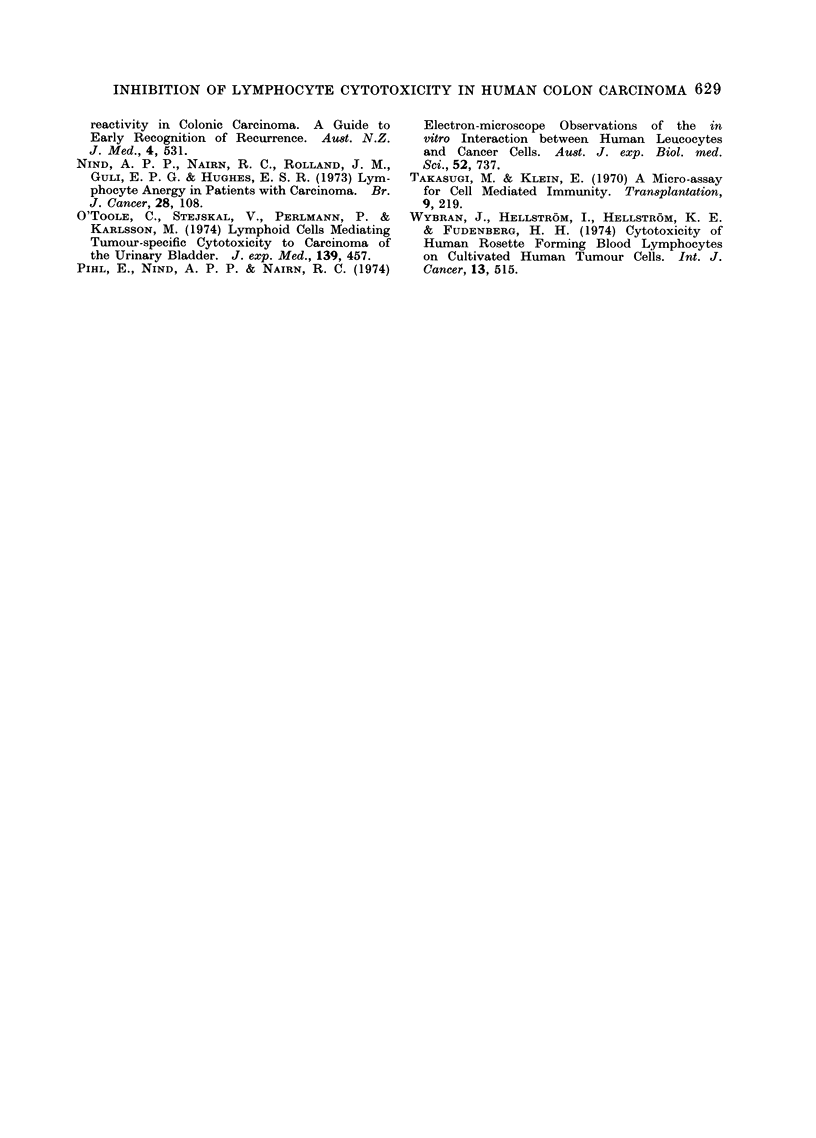# Analysis of inhibition of lymphocyte cytotoxicity in human colon carcinoma.

**DOI:** 10.1038/bjc.1975.108

**Published:** 1975-06

**Authors:** A. P. Nind, N. Matthews, E. A. Pihl, J. M. Rolland, R. C. Nairn

## Abstract

Serum inhibition of autochthonous lymphocyte cytotoxicity for tumour cells has been studied in 112 cases of colonic carcinoma. Addition of patient's serum to the lymphocyte tumour cell reaction mixture resulted in decreased cytotoxic reactivity of lymphocytes from 8 of 39 cytotoxic positive cases. It was also shown that sera could inhibit if separately preincubated with the lymphocytes (4 cases) or the target cells (2 cases). A tumour antigen preparation inhibited only when incubated with the lymphocytes. Inhibition by serum or antigen appeared to be specific for colon carcinoma. Four cases were specially studied to determine the mode of lymphocyte killing of tumour cells: in 3 it was mediated largely if not entirely by T lymphocytes, and in the fourth by both T and non-T cells. The findings support the view that T lymphocytes lose their anti-tumour reactivity in vivo in the presence of circulating antigen or antigen-antibody complexes such as would occur with progressive tumour growth.


*Br. J. Cancer* (1975) **31**, 620

### ANALYSIS OF INHIBITION OF LYMPHOCYTE CYTOTOXICITY IN HUMAN COLON CARCINOMA

A. P. P. NIND, N. MATTHEWS, E. A. V. PIHL, J. M. ROLLAND AND R. C. NAIRN

*From the Department of Pathology and Immunology, Monash University Medical School, Melbourne, Australia*

Received 6 January 1975. Accepted 24 February 1975

**Summary.—Serum inhibition of autochthonous lymphocyte cytotoxicity for tumour cells has been studied in 112 cases of colonic carcinoma. Addition of patient's serum to the lymphocyte tumour cell reaction mixture resulted in decreased cytotoxic reactivity of lymphocytes from 8 of 39 cytotoxic positive cases. It was also shown that sera could inhibit if separately preincubated with the lymphocytes (4 cases) or the target cells (2 cases). A tumour antigen preparation inhibited only when incubated with the lymphocytes. Inhibition by serum or antigen appeared to be specific for colon carcinoma. Four cases were specially studied to determine the mode of lymphocyte killing of tumour cells: in 3 it was mediated largely if not entirely by T lymphocytes, and in the fourth by both T and non-T cells. The findings support the view that T lymphocytes lose their anti-tumour reactivity *in vivo* in the presence of circulating antigen or antigen–antibody complexes such as would occur with progressive tumour growth.**

IN PREVIOUS reports of lymphocyte anergy in patients with carcinoma (Nind *et al.*, 1973), it was speculated that the observed *in vitro* inactivity of the local lymphocyte populations might be due to *in vivo* inactivation of specific effector cells by free tumour antigen or complexes of tumour antigen with tumour directed antibody. This would explain the decrease in leucocyte cytotoxicity and the increase in serum inhibitory effect that follows progressive growth or regrowth of tumour (Nairn *et al.*, 1974). Study of the phenomenon of inhibition of leucocyte cytotoxicity has hitherto been hampered by ignorance of the particular effector cell type when testing *in vitro*. We have now obtained inhibition of cytotoxicity by pretreatment of effector or target cell populations with sera of colon carcinoma patients and have also prepared a tumour antigen extract capable of inactivating the effector cells. The reactions in limited cross-reactivity tests with other tumour systems appeared to be specific for colon carcinoma. Fractionation studies in 4 cases showed the lymphocyte class cytotoxic for the colonic carcinoma cells to be predominantly T cell.

### MATERIALS AND METHODS

Specimen and blood collection were as described previously (Nairn *et al.*, 1971; Nind *et al.*, 1973). Tumours, lymph nodes adjacent to the tumour, and in 2 cases spleen removed at operation, were teased gently into culture medium 199 containing 10% foetal calf serum (hereafter referred to as the culture medium). The resultant suspensions (tumour cells washed twice, spleen and lymph node cells washed 7 times with culture medium) were used fresh for immediate testing and any residue was stored with 10% dimethylsulphoxide in liquid nitrogen for later use. Lymphocytes were obtained from heparinized blood samples by sedimentation of the erythrocytes at 37°C and washing the leucocyte-rich supernatant plasma through a 2 cm glass wool column with culture medium. The lymphocyte eluate, which was contaminated by less than 10% granulocytes, was washed 7 times with culture medium before use.

Serum obtained from clotted blood samples from each patient was complement inactivated at 56°C for 30 min.

*Lymphocyte cytotoxicity testing.*—This was performed quantitatively in Falcon 3034 microtest plates by a modification of the method of Takasugi and Klein (1970) (Pihl, Nind and Nairn, 1974). Tumour cell suspensions, adjusted to $10^5$ cells/ml in culture medium were micropipetted in 10 $\mu$l volumes into each well. Plates were incubated for 24 h at 37°C in a moist atmosphere of 5% $CO_2$ in air. They were then washed 3 times with culture medium to remove non-adhered tumour cells. Those remaining in each well were counted and lymphocytes were added at a ratio of 200 to 1 tumour cell, in rows of 5 or 6 replicate wells for each experiment. The culture plate was reincubated for a further 48 h, washed gently 3 times with physiological saline at 37°C, and the remaining adherent cells were fixed with methanol for 10 min and counted under water using an inverted phase-contrast microscope. Test blood lymphocytes were accompanied by a row of homologous control blood lymphocytes obtained from 1 of 20 normal healthy adults of either sex. Control splenocytes were from a splenectomized patient who had a small leiomyosarcoma of the stomach. Cytotoxicity was expressed as

$$\frac{nC - nT}{nC} \cdot 100$$

where $nC$ is the mean number of tumour cells remaining adherent in the control wells and $nT$ the mean number remaining in the test wells. Student's $t$ test was performed for each experiment and a difference between cytotoxicity means at the $P < 0{\cdot}05$ level was regarded as significant.

*Serum inhibition of lymphocyte cytotoxicity.*—This was tested in 112 cases (39 of which were positive for autochthonous lymphocyte cytotoxicity by blood, lymph node or spleen) by the incorporation of 5% patient's serum in the lymphocyte suspension immediately before adding to the target cells. As controls, 5% normal group AB serum was added to patient's lymphocytes, and each serum was tested on homologous normal lymphocytes. Inhibition was expressed as a percentage according to the formula

$$\frac{C_{AB} - C_P}{C_{AB}} \cdot 100$$

where $C_{AB}$ was the cytotoxicity of the patient's lymphocytes with normal group AB serum and $C_P$ their cytotoxicity with the patient's serum. Student's $t$ test was used to compare the cell counts with test and control sera and a difference between means at the $P < 0{\cdot}05$ level was regarded as significant inhibition.

*Preincubation of lymphocytes with serum.*—Three sera (Cases 1, 2, 4) with strong autochthonous inhibition by the routine test were also tested in homologous colonic carcinoma/lymphocyte systems (Cases 1, 3, 6, 32, 37) (see Table I). One or 2 of these 3 sera were preincubated with the lymphocytes of each of the 5 cases before addition to target tumour cells. This was performed by adding to 5 $\mu$l of test serum in a polystyrene Durham tube, 0·1 ml of lymphocyte suspension, usually containing $2 \times 10^6$/ml, adjusted where necessary to provide the usual ratio of lymphocytes to tumour cells of 200 : 1. After mixing, the tube was incubated at 37°C in a moist atmosphere of 5% $CO_2$ in air for 45 min. The cells were then washed twice with culture medium by centrifugation at 500 $g_{max}$ for 5 min and resuspended in 0·1 ml of culture medium; 10 $\mu$l aliquots were added to target cell wells for cytotoxicity testing in the usual way. Inhibition was determined using similar controls and the same calculation procedure as for the routine serum inhibition test.

*Specificity of serum inhibition.*—Cross-reactive inhibition tests by serum from colonic carcinoma patients were performed with cytotoxic lymphocytes and target cells from different colonic carcinomata, carcinomata of the stomach and malignant melanomata. By the routine technique, inhibitory sera from up to 3 colonic carcinomata were tested by addition to the lymphocyte tumour systems of 3 other homologous colonic carcinomata, 1 gastric carcinoma and 3 melanomata. Autochthonous inhibitory sera from 1 gastric carcinoma and 2 melanomata were examined in cytotoxicity tests of 2 autochthonous lymphocyte positive colonic carcinoma cases. The specificity of inhibitory sera preincubated with lymphocytes was examined for up to 3 colonic carcinoma sera tested with the lymphocytes of each of 5 homologous colonic carcinomata, 2 gastric carcinomata and 2 melanomata reacted against their autochthonous tumours.

Autochthonous inhibitory sera from 1 gastric carcinoma and 2 melanoma patients were preincubated with the lymphocytes of each of 3 colonic carcinoma cases and the lymphocytes then tested against their respective tumours.

*Preincubation of lymphocytes with antigen.*—Homogenized and de-fatted tissue from a colonic carcinoma (Case 1) was acid extracted by the method of Dickinson, Caspary and Field (1973), dialysed against water and freeze-dried for use as required. A similar extract was prepared from 10 pooled normal colon mucosas. Any inhibitory activity of the preparations was determined by preincubating at concentrations of 5, $5 \times 10^{-1}$, $5 \times 10^{-2}$, $5 \times 10^{-3}$ and $5 \times 10^{-4}$ $\mu g$ dry weight of antigen per ml with the splenocytes from Case 1, which were then washed twice and tested for cytotoxicity by adding to Case 1 tumour cells in microculture plates. In control cytotoxicity experiments, equivalent concentrations of either antigen were preincubated with homologous normal lymphocytes or splenocytes. Specificity of inhibition by the tumour antigen was investigated by preincubating the extract at the same concentrations with lymphocytes from one case each of carcinoma of the stomach and melanoma before they were reacted with their respective tumour cells. In further experiments lymphocytes from 4 other colonic carcinoma cases, one other gastric carcinoma and one other melanoma were pretreated with 0·5 $\mu g$/ml of either antigen.

*Preincubation of lymphocytes with antigen plus serum.*—To determine if serum and antigen effects were additive, Case 1 serum, which inhibited 4 of 5 colonic carcinoma tests, was combined with 0·5 $\mu g$/ml of the tumour antigen extract, which gave alone about 50% inhibition. The mixture was preincubated with the lymphocytes of the same 5 cases, which were then tested for cytotoxicity against their respective targets and compared with lymphocytes pretreated with serum or tumour antigen alone.

*Preincubation of target tumour cells with antigen and serum.*—The tumour cells of 2 cases (Case 1, 6) were preincubated with tumour antigen, inhibitory serum or both together. Replicate tumour cell microcultures were incubated at 37°C for 45 min in a moist atmosphere of 5% $CO_2$ in air with 0·01 ml aliquots of (a) 5% inhibitory serum, (b) 0·5 $\mu g$/ml of tumour antigen or (c) a mixture of both, each at the same concentration as in (a) or (b). The preparations were then washed twice with culture medium and the relevant lymphocytes were added in the usual ratio of 200 : 1 tumour cell. Comparison was made with tests against untreated target cells.

*Fractionation of lymphocyte populations by rosette formation.*—Human lymphocytes forming rosettes with sheep erythrocytes have the characteristics of T cells (Coombs *et al.*, 1970; Jondal, Holm and Wigzell, 1972). Lymphocyte preparations purified by Hypaque–Ficol sedimentation were incubated with sheep erythrocytes for 1 h at 37°C and again centrifuged in Hypaque–Ficol (Wybran *et al.*, 1974). The non-rosette forming cells at the interface and the rosetted cells in the pellet were collected carefully and counted. The cells were washed twice and resuspended in culture medium at the appropriate concentration for cytotoxicity testing (usually $2 \times 10^6$/ml). Sheep erythrocytes were added to the unfractionated and non-rosette forming populations to make the cell density the same as in the rosette forming fraction. Aliquots of either fraction and of the unfractionated cells were added to microcultures of target cells for cytotoxicity testing in a minimum of 10 replicate wells to give at least 18 degrees of freedom for each test of significance. In 2 experiments, normal homologous lymphocyte populations (one spleen, one blood) were fractionated simultaneously with the test lymphocytes to provide cytotoxicity controls. In another 3 experiments, where this was not done, the test preparation was compared with an unfractionated control. Preliminary experiments with normal blood lymphocytes showed that the fractionation did not confer cytotoxicity on non-cytotoxic unfractionated cells.

### RESULTS

### *Serum inhibition of lymphocyte cytotoxicity*

Eight of the 39 (21%) lymphocyte cytotoxic cases showed significant reduction of cytotoxicity by their serum added to the lymphocyte/tumour cell mixture (Table I). Three of the inhibitory sera tested (Cases 1, 2, 4) produced significant inhibition with 3 other colonic carcinoma systems but only slight inhibition in

TABLE I.—*Results of Serum Inhibition Tests in Lymphocyte Cytotoxic-positive Cases of Colonic Carcinoma*

| Case No. | Tumour cell no./well: Patient's lymphocytes: Patient's serum | Patient's lymphocytes: Normal serum | Control lymphocytes: Patient's serum | Control lymphocytes: Normal serum | % Inhibition | Degrees of freedom | *t* value |
|---|---|---|---|---|---|---|---|
| 1 (73/142) | 20±2 | 15±3 | 19±2 | 22±3 | 100 | 8 | 3·1* |
| 2 (73/115) | 17±6 | 6±2 | 14±6 | 15±4 | 100 | 8 | 3·9** |
| 3 (74/113) | 31±2 | 23±2 | 30±4 | 29±3 | 100 | 8 | 6·3*** |
| 4 (73/163) | 29±4 | 21±4 | 31±3 | 30±4 | 80 | 10 | 3·5** |
| 5 (74/87) | 21±2 | 18±2 | 23±2 | 23±2 | 59 | 8 | 2·4* |
| 6 (74/139)† | 26±5 | 24±5 | 29±6 | 31±8 | 57 | 8 | 0·6 |
| 7 (73/83)‡ | 35±3 | 23±2 | 53±4 | 51±9 | 38 | 8 | 7·4*** |
| 8 (74/12) | 18±2 | 17±4 | 22±1 | 24±2 | 38 | 10 | 0·4 |
| 9 (74/120)‡ | 19±2 | 15±2 | 25±4 | 26±5 | 36 | 8 | 3·2* |
| 10 (74/102) | 21±2 | 15±2 | 34±1 | 35±9 | 33 | 8 | 4·7** |
| 11 (74/121) | 21±5 | 17±4 | 30±6 | 28±3 | 23 | 8 | 1·4 |
| 12 (74/81)‡ | 25±6 | 25±3 | 30±3 | 32±1 | 22 | 8 | 0 |
| 13 (74/137) | 21±3 | 20±2 | 25±3 | 25±2 | 20 | 10 | 0·7 |
| 14 (74/54) | 13±2 | 15±2 | 17±3 | 21±5 | 17 | 10 | 1·7 |
| 15 (74/77) | 27±3 | 26±3 | 40±2 | 41±1 | 11 | 8 | 0·5 |
| 16 (73/130) | 23±3 | 21±3 | 45±2 | 47±2 | 11 | 10 | 1·1 |
| 17 (73/70) | 28±4 | 25±5 | 67±5 | 68±7 | 8 | 10 | 1·1 |
| 18 (74/48) | 8±1 | 7±2 | 36±2 | 38±3 | 5 | 10 | 1·1 |
| 19 (74/50) | 47±5 | 44±5 | 107±7 | 105±13 | 3 | 10 | 1·0 |
| 20 (73/134) | 4±1 | 4±1 | 28±4 | 27±6 | 1 | 10 | 0 |
| 21 (73/53) | 39±8 | 45±7 | 62±9 | 53±12 | 0 | 18 | 1·8 |
| 22 (73/72) | 27±5 | 30±2 | 40±6 | 45±5 | 0 | 10 | 1·4 |
| 23 (73/92) | 38±7 | 48±9 | 63±5 | 65±3 | 0 | 10 | 2·1 |
| 24 (73/96) | 10±3 | 11±5 | 45±5 | 44±4 | 0 | 9 | 0·4 |
| 25 (73/106) | 12±3 | 13±3 | 20±2 | 20±3 | 0 | 8 | 0·5 |
| 26 (73/138) | 11±2 | 15±5 | 43±2 | 42±4 | 0 | 10 | 1·8 |
| 27 (73/141) | 4±1 | 6±2 | 37±4 | 37±3 | 0 | 10 | 2·2 |
| 28 (74/3) | 14±4 | 19±4 | 32±8 | 31±4 | 0 | 9 | 1·5 |
| 29 (74/13) | 9±2 | 11±3 | 20±2 | 20±2 | 0 | 10 | 1·4 |
| 30 (74/26) | 16±2 | 20±5 | 30±3 | 30±3 | 0 | 10 | 1·8 |
| 31 (74/32) | 15±9 | 18±9 | 28±5 | 30±5 | 0 | 10 | 0·6 |
| 32 (74/43) | 18±6 | 21±2 | 33±2 | 33±5 | 0 | 10 | 1·2 |
| 33 (74/64) | 12±2 | 12±1 | 23±3 | 23±2 | 0 | 10 | 0 |
| 34 (74/70) | 20±3 | 23±2 | 29±3 | 28±2 | 0 | 10 | 2·0 |
| 35 (74/92) | 33±15 | 34±7 | 61±8 | 60±4 | 0 | 10 | 0·1 |
| 36 (74/94) | 18±2 | 19±2 | 25±3 | 24±3 | 0 | 10 | 0·9 |
| 37 (74/104) | 20±4 | 22±3 | 33±2 | 32±3 | 0 | 10 | 1·0 |
| 38 (74/112)‡ | 10±3 | 11±3 | 23±3 | 25±2 | 0 | 8 | 0·5 |
| 39 (74/132) | 12±3 | 12±5 | 20±1 | 20±2 | 0 | 8 | 0 |

* $0{\cdot}05 > P > 0{\cdot}01$. ** $0{\cdot}01 > P > 0{\cdot}001$. *** $P < 0{\cdot}001$.

† Tested by splenocytes with homologous splenocytes as control.

‡ Tested by lymph node lymphocytes with homologous blood lymphocytes as control. All other tests with blood lymphocytes.

carcinoma of the stomach and no inhibition in 3 melanomata. Autochthonous inhibitory sera from 2 patients with melanoma and 1 with carcinoma of the stomach were not inhibitory when added to 2 colonic carcinoma cytotoxic systems (Cases 1 and 15).

*Preincubation of lymphocytes with antigen, serum or both together*

Pretreatment of the reactive splenocytes from Case 1 with increasing concentrations of the colonic carcinoma antigen resulted in a gradual decrease in cytotoxicity (Figure). There was no significant inhibition by normal colon antigen. The colonic tumour antigen at the same concentrations did not inhibit the lymphocytes of a patient with carcinoma of stomach (Case 40) or melanoma (Case 42). Pretreatment with 0·5 μg/ml colonic carcinoma antigen reduced cytotoxicity by lymphocytes of 3 (Cases 3, 32, 37) of 4 cases of colonic carcinoma (see

FIG.—Effect on autochthonous lymphocyte anti-tumour cytotoxicity of preincubating lymphocytes with preparations of colonic carcinoma or normal colon at different concentrations.

TABLE II.—*Effect of Preincubation of Cytotoxic Lymphocytes with Inhibitory Serum and Antigen Preparations*

| Lymphocytes† Case no. | Tumour Case no. | % Inhibition with: Tumour antigen 5 μg/ml | Tumour antigen 0·5 μg/ml | Normal colon antigen 5 μg/ml | Normal colon antigen 0·5 μg/ml | 5% serum of Case 1 | 5% serum of Case 1 + Tumour antigen 0·5 μg/ml |
|---|---|---|---|---|---|---|---|
| *Ca Colon* | | | | | | | |
| 1 | 1 | 99** | 54** | 27 | 24 | 95‡ | 0 |
| 32 | 6 | .. | 48* | .. | 0 | 48* | 0 |
| 6 | 6 | 0 | 0 | 0 | 0 | 81** | 75* |
| 37 | 37 | 100** | 60* | 0 | 0 | 46* | 100** |
| 3 | 3 | 100** | 100* | .. | 24 | 6 | 30* |
| *Ca Stomach* | | | | | | | |
| 40 | 40 | 0 | 10 | 0 | 0 | 24 | .. |
| 41 | 41 | .. | 0 | .. | 0 | 0 | 0 |
| *Melanoma* | | | | | | | |
| 42 | 42 | 9 | 0 | 8 | 0 | 23 | .. |
| 43 | 43 | 0 | .. | 0 | .. | 5 | .. |

† Untreated lymphocyte cytotoxicity values ranged from 25% to 44% and all were significant.
.. Not done.
* $0\cdot05 > P > 0\cdot01$; ** $0\cdot01 > P > 0\cdot001$.

Table II). Case 6 showed no inhibition nor did 1 carcinoma of stomach (Case 41) and 1 melanoma (Case 43). The same concentration of normal colon extract did not inhibit any of the 5 colonic carcinoma lymphocyte preparations.

Preincubation of lymphocytes with serum resulted in 4 of the 5 colonic carcinoma cases tested showing reduction of lymphocyte cytotoxicity with at least 1 of the 3 sera. The results obtained with the serum of Case 1 are summarized in Table II. Case 3, which showed no inhibition by serum from Case 1, was not tested with the other 2 sera. The serum from Case 1 did not significantly inhibit when preincubated with lymphocytes in 2 positive gastric carcinoma systems (Cases 40, 41) and 2 melanomata (Cases 42, 43). Autochthonous inhibitory sera, detected by the same technique, from 1 gastric carcinoma case and 2 melanomata did not interfere with the lymphocyte reactivity of any of 3 colon carcinomata (Cases 1, 32, 36).

The results of preincubating with the mixture of antigen plus serum, summarized also in Table II, were not uniform in the 5 cases studied. Where preincubation with either the serum or antigen separately caused inhibition, in Case 37, the mixture gave increased inhibition while in Cases 1 and 32 there was no change. Where only antigen reduced cytotoxicity (Case 3), addition of the inhibitory sera abrogated this effect. There was no change in serum inhibition in Case 6 by adding tumour antigen.

Additional data from separate experiments (Table III) make comparisons between lymphocyte and target cell preincubations. The difference in the percentage cytotoxicity of similar preparations in Tables II and III are within the limits of experimental variation.

*Preincubation of target tumour cells with antigen, serum or both together*

The colonic carcinoma antigen preparation, as might be expected, had no effect on the tumour cells (Table III). Inhibitory serum blocked tumour cells as targets whether tested against autochthonous or homologous lymphocytes. Combination of serum with the antigen preparation studied in Cases 1 and 6 resulted in abrogation of serum blocking.

TABLE III.—*Comparison of Effect of Preincubation of Target Tumour Cells and Cytotoxic Lymphocytes with Antigen and Inhibitory Serum*

| | | % Blocking or inhibition | | | | | |
|---|---|---|---|---|---|---|---|
| | | Tumour antigen† preincubated with | | Serum‡ preincubated with | | Tumour antigen† + serum‡ preincubated with | |
| Lymphocytes* Case no. | Tumour Case no. | Lymphocytes | Tumour | Lymphocytes | Tumour | Lymphocytes | Tumour |
| 1 | 1 | 96** | 6 | 100** | 30** | 18 | 0 |
| 1 | 6 | 40** | 9 | 54** | 60** | 0 | 5 |
| 6 | 1 | 29 | 0 | 79** | 0 | 61*** | 0 |
| 6 | 6 | 0 | 0 | 81** | 49** | 75*** | 23 |

* Untreated lymphocyte cytotoxicity values ranged from 46 % to 49 % for Case 1 and 35 % to 38 % for Case 6; all were significant.

† 0·5 μg/ml.

‡ 5 % serum from Case 1.

** $0 \cdot 05 > P > 0 \cdot 01$; *** $0 \cdot 01 > P > 0 \cdot 001$.

*Fractionation of lymphocyte populations by rosette formation*

The results of the 5 cytotoxic positive cases studied by these procedures are summarized in Table IV. The rosette-rich fraction of 4 of 5 cases showed cytotoxicity to the tumour, while the non-rosette forming fraction was cytotoxic in only one of the 5 (Case 39); in Case 37, the cytotoxicity was lost by fractionation. This last was possibly due to a requirement for co-operation by both cell classes. None of the control homologous lymphocyte fractions showed cytotoxicity. Cases 1 and 6 were tested with frozen–thawed splenocyte preparations, and because the Hypaque–Ficol centrifugation tended to concentrate dead cells in the pellet, the rosette counts are lower than would otherwise be expected. However, this does not invalidate the results because neither case showed cytotoxicity in the non-rosette forming fraction in which the proportion of viable cells had been selectively increased.

### DISCUSSION

Investigations in 5 cases suggest that human *in vitro* anti-colon carcinoma lymphocyte cytotoxicity is mostly a T cell phenomenon. This is in accord with similar observations of Wybran *et al.* (1974) in melanoma patients. It is not a general phenomenon, as indicated by O'Toole *et al.*'s (1974) report of predominantly non-T cell cytotoxicity in urinary bladder carcinoma. Indeed, we ourselves have evidence (Nairn *et al.*, 1975) of change of killer cell type in our *in vitro* test applied serially to a case of melanoma as tumour growth progressed. Similar findings in murine tumours are reported by Lamon *et al.* (1973).

The effects of serum factors and antigens on lymphocyte anti-tumour immunoreactivity in man have been reported by Baldwin, Embleton and Price (1973) and Hellström *et al.* (1973). Our study in a large series of colonic carcinoma patients of the influence of serum factors on the apparent predominantly T lymphocyte cytotoxicity has shown inhibition by 21% of sera from autochthonous cytotoxic positive cases (*i.e.* 7% of all 112 colon carcinoma cases tested). The normal control lymphocytes obtained from 20 normal individuals were cytotoxic by our technique in only 2 of the 112 tests performed. In these 2 tests, lymphocytes from 2 individuals on one occasion each showed 40% and 43% cytotoxicity compared with a blank reading without lymphocytes added.

The inhibitory effects were associated with factors operating on the effector lymphocytes and on the target tumour

TABLE IV.—*Effect on Lymphocyte Cytotoxicity of Separation into Rosette Forming and Non-Rosette Forming Fractions*

| | | Unfractionated | | Non-rosette forming | | | Rosette-forming | | |
|---|---|---|---|---|---|---|---|---|---|
| Lymphocytes | Target tumour Case no. | Target cells/well ±SD | % Cytotoxicity | Target cells/well ± SD | % Cytotoxicity | % Rosettes | Target cells/well ± SD | % Cytotoxicity | % Rosettes |
| Case 1 (spleen) | 1 | 23·9±4·3 | 20† | 26·3±4·3 | 9 | 0 | 20·3±3·5 | 35*** | 16* |
| Control (spleen) | 1 | 29·7±3·7 | | 29·0±4·7 | | 3 | 31·0±4·3 | | 45* |
| Case 32 (blood) | 6 | 11·3±3·9 | 60*** | 25·3±2·0 | 12 | 5 | 14·8±1·6 | 48*** | 80 |
| Control (blood) | 6 | 28·6±4·6 | | | | | | | |
| Case 6 (spleen) | 6 | 19·8±2·6 | 31† | 26·7±4·8 | 7 | 6 | 18·0±5·0 | 37*** | 33* |
| Control (blood) | 6 | 28·6±4·6 | | | | | | | |
| Case 39 (blood) | 39 | 10·0±2·3 | 50*** | 8·3±2·9 | 58*** | 0 | 14·1±2·8 | 30*** | 89 |
| Control (blood) | 39 | 20·1±2·1 | | | | | | | |
| Case 37 (blood) | 37 | 13·4±3·7 | 45*** | 19·7±4·3 | 2 | 0 | 19·7±3·5 | 12 | 88 |
| Control (blood) | 37 | 24·3±5·3 | | 20·1±5·1 | | 5 | 22·5±5·9 | | 77 |

* Low values for splenocytes due to their reduced viability.

† $0 \cdot 05 > P > 0 \cdot 01$; ** $0 \cdot 01 > P > 0 \cdot 001$; *** $P < 0 \cdot 001$.

cells. Preincubation of reactive lymphocytes either with serum or the tumour antigen extract inhibited cytotoxicity in most colonic carcinoma cases tested. Thus, it would seem that lymphocytes sensitized to tumour antigens are able to bind soluble antigenic material, thereby becoming unreactive to the target tumour cell surface. Serum inhibited both the lymphocytes and target cells suggesting the presence of antigen–antibody complexes at or near equivalence. Greater avidity of lymphocyte surface receptors for antigen could result in binding of the complex to the lymphocyte by dissociation of lower avidity antibody in the complex. Similarly, antibody in the complex might be expected to have greater affinity for antigen on tumour cells and result in cross-linking of complex on the tumour cell surface. Combination of serum and the antigen extract consistently showed no blocking when preincubated with tumour cells, presumably because of interference by a high concentration of soluble tumour antigen. However, preincubation of lymphocytes with serum and antigen together sometimes had different effects from either component alone.

Serum abrogation of the *in vitro* cytotoxicity in our tests appears to be tumour determined because there was no cross-reaction with unlike carcinomata. The specificity of inhibitory tumour antigen is in contrast with the observations of Dickinson *et al.* (1973); they found with the macrophage electrophoretic mobility (MEM) test that antigen material prepared by the same method cross-reacted with all tumour types. The difference may be attributable to the lymphocyte cytotoxicity being a functional effector test, possibly less sensitive but more specific than MEM, which is a reactor response perhaps capable of being provoked by a broader spectrum of antigens. The tumour type specificity does not support nonspecific explanations of inactivation *in vivo*. The apparent quantitative inhibition by antigen of the effector cells is consistent with the view that progressive growth of tumour, by increasing concentration of soluble tumour antigen in the circulation and acting either alone or complexed with antibody, can impair the effector responsiveness *in vivo* of anti-tumour lymphocytes.

This work was supported by grants from the Anti-Cancer Council of Victoria and the National Health and Medical Research Council. For providing us with specimens we thank Professor E. S. R. Hughes, Mr A. J. Rollo and Mr A. M. Cuthbertson. We also thank Mrs K. Chism, Miss L. Jean-Germaine and Mr T. Wilson for technical assistance.

### REFERENCES

Baldwin, R. W., Embleton, M. J. & Price, M. R. (1973) Inhibition of Lymphocyte Cytotoxicity for Human Colon Carcinoma by Treatment with Solubilized Tumour Membrane Fractions. *Int. J. Cancer*, **12**, 84.

Coombs, R. R. A., Gurner, B. W., Wilson, A. B., Holm, G. & Lindgren, B. (1970) Rosette-formation between Human Lymphocytes and Sheep Red Cells not Involving Immunoglobulin Receptors. *Int. Archs Allergy*, **39**, 658.

Dickinson, J. P., Caspary, E. A. & Field, E. J. (1973) A Common Tumour Specific Antigen. *Br. J. Cancer*, **27**, 99.

Hellström, I., Warner, G. A., Hellström, K. E. & Sjögren, H. O. (1973) Sequential Studies on Cell-mediated Tumor Immunity and Blocking Serum Activity in Ten Patients with Malignant Melanoma. *Int. J. Cancer*, **11**, 280.

Jondal, M., Holm, G. & Wigzell, H. (1972) Surface Markers on Human T and B Lymphocytes. I. A Large Population of Lymphocytes Forming Non-Immune Rosettes with Sheep Red Blood Cells. *J. exp. Med.*, **136**, 207.

Lamon, E. W., Wigzell, H., Klein, E., Andersson, B. & Skurzak, H. M. (1973) The Lymphocyte Response to Primary Moloney Sarcoma Virus Tumors in Balb/c Mice. Definition of the Active Subpopulations at different Times after Infection. *J. exp. Med.*, **137**, 1472.

Nairn, R. C., Nind, A. P. P., Guli, E. P. G., Davies, D. J., Rolland, J. M., McGiven, A. R. & Hughes, E. S. R. (1971) Immunological Reactivity in Patients with Carcinoma of Colon. *Br. med. J.*, iv, 706.

Nairn, R. C., Nind, A. P. P., Pihl, E., Rolland, J. M. & Matthews, N. (1975) Immunological Anergy in Melanoma. In *Proc. 9th Internat. Pigment Cell Conference*. Ed. V. T. Riley. Basel: Karger. (In press.)

Nairn, R. C., Rollo, A. J., Nind, A. P. P., Rolland, J. M., Guli, E. P. G., Pihl, E. A. V. & Stevens, D. P. (1974) Anti-Tumour Immunoreactivity in Colonic Carcinoma. A Guide to Early Recognition of Recurrence. *Aust. N.Z. J. Med.*, **4**, 531.

NIND, A. P. P., NAIRN, R. C., ROLLAND, J. M., GULI, E. P. G. & HUGHES, E. S. R. (1973) Lymphocyte Anergy in Patients with Carcinoma. *Br. J. Cancer*, **28**, 108.

O'TOOLE, C., STEJSKAL, V., PERLMANN, P. & KARLSSON, M. (1974) Lymphoid Cells Mediating Tumour-specific Cytotoxicity to Carcinoma of the Urinary Bladder. *J. exp. Med.*, **139**, 457.

PIHL, E., NIND, A. P. P. & NAIRN, R. C. (1974) Electron-microscope Observations of the *in vitro* Interaction between Human Leucocytes and Cancer Cells. *Aust. J. exp. Biol. med. Sci.*, **52**, 737.

TAKASUGI, M. & KLEIN, E. (1970) A Micro-assay for Cell Mediated Immunity. *Transplantation*, **9**, 219.

WYBRAN, J., HELLSTRÖM, I., HELLSTRÖM, K. E. & FUDENBERG, H. H. (1974) Cytotoxicity of Human Rosette Forming Blood Lymphocytes on Cultivated Human Tumour Cells. *Int. J. Cancer*, **13**, 515.